# Could pentraxin-3 be a new marker for subclinical inflammation in familial Mediterranean fever?

**DOI:** 10.1186/1546-0096-13-S1-P98

**Published:** 2015-09-28

**Authors:** S Yüksel, E Karadağlı, H Evrengül, H Şenol

**Affiliations:** 1 Pamukkale University, School of Medicine, Pediatric Rheumatology, Denizli, Turkey; 2 Pamukkale University, School of Medicine, Biostatistics, Denizli, Turkey

## Introduction

Pentraxin-3 (PTX-3) is a long pentraxin that is structurally related to the short pentraxins as C-reactive protein (CRP). It is known to play an important role in innate immunity and inflammatory regulation. CRP and serum amyloid A (SAA) are sensitive and reliable markers of inflammation in FMF attack as well as chronic and subclinical inflammation during attack-free period. To date, there is no information about PTX-3 in FMF inflammation.

## Aim

The aim of the study was to investigate the progress of serum PTX-3 levels together with traditional acute phase reactants in FMF patients during attack and attack free period (two weeks after the attack) and also assess whether PTX-3 could be related with subclinical inflammation.

## Material and method

A prospective cross-sectional study was conducted between June 2013 and July 2014. A total of 45 consecutive children with FMF who were diagnosed according to the Tel-Hashomer and Yalçınkaya criteria were enrolled during the attack period. Blood samples were obtained from the patients during attack and attack free period (two weeks after the attack) and healthy children who were matched in terms of age and sex.

## Results

The study group consisted of 45 children with FMF (24 boys, 21 girls, mean age 9.5±3.8 years) and 40 healthy children. In FMF patients attack white blood cell (WBC) count, CRP, erythrocyte sedimentation rate (ESR), fibrinogen, SAA and PTX-3 levels were significantly higher than attack-free period and healthy subjects. In attack-free period, there were no significant differences between patients and healthy children in terms of WBC, CRP levels. Although mean attack-free period ESR, fibrinogen and SAA levels were higher than the controls, those markers were within the normal range. Whereas, mean attack-free PTX-3 level was still significantly higher than controls.

**Table 1:** 

	During Attack (Mean±SD)	After Attack (Mean±SD)	Controls (Mean±SD)	*P_1_*	*P_2_*	*P_3_*
**WBC (x10^9^/L)**	12.1±4.6	8.1±2.5	8.2±2.5	***<0.001***	***<0.001***	*>0.05*

**MPV (fL)**	7.5±0.70	7.6±0.58	7.3±0.57	*>0.05*	*>0.05*	*>0.05*

**CRP (mg/dL) (normal: 0-1)**	4.7±4.2	0.15±0.19	0.11±0.15	***<0.001***	***<0.001***	*>0.05*

**ESR (mm/hour) (normal < 20)**	44.1±20.8	13.9±7.24	6.6±4.5	***<0.001***	***<0.001***	***<0.001***

**Fibrinogen (mg/dL) (normal: 180-350)**	371.0±70.9	257.0±55.3	220±45	***<0.001***	***<0.001***	***<0.001***

**SAA (mg/L) (normal: 0-7)**	306.5±283.1	5.5±3.8	4.1±1.2	***<0.001***	***<0.001***	***0.032***

**Pentraxin-3 (ng/mL)**	3.2±0.6	1.89±0.14	0.87±0.38	***<0.001***	***<0.001***	***<0.001***

*P_1_*: During attack vs after attack, *P*_2_: During attack vs controls, *P*_3_: After attack vs controls

## Conclusion

Serum PTX-3 levels increased during the attacks of FMF and decreased during the attack free period however mean level of it was still higher than healthy subjects. We suggest that PTX-3 might be a new marker for both attack period and subclinical inflammation in FMF patients.

